# The Very Low IgE Producer: Allergology, Genetics, Immunodeficiencies, and Oncology

**DOI:** 10.3390/biomedicines11051378

**Published:** 2023-05-06

**Authors:** Paolo Maria Matricardi

**Affiliations:** Charité—Universitätsmedizin Berlin, Corporate Member of Freie Universität Berlin and Humboldt-Universität zu Berlin, Department of Pediatric Respiratory Medicine, Immunology and Critical Care Medicine, Augustenburger Platz 1, 13353 Berlin, Germany; paolo.matricardi@charite.de

**Keywords:** allergo-oncology, allergy, atopy, immunodeficiency, immunoglobulin E, normal values

## Abstract

Opposite to other immunoglobulin (Ig) classes and subclasses, there is no consensus on the definition of normal levels of serum total IgE. However, longitudinal studies on birth cohorts produced growth charts of total IgE levels in helminth-free and never atopic children and defining the normal ranges of total serum IgE concentration at the individual, rather than population, level. Accordingly, very ‘low IgE producers’ (i.e., children whose tIgE level belong to the lowest percentiles) became atopic while keeping their total IgE levels in a range considered ‘normal’ if compared to the general age-matched population but ‘abnormally high’ if projected on the tIgE growth chart against the trajectory of that child’s own percentile levels. In ‘low IgE producers’, the IgE-specific activity, i.e., the ratio between allergen-specific and total IgE, is more important than the absolute specific IgE levels to confirm causality between allergen exposure and allergic symptoms. Patients with allergic rhinitis or peanut anaphylaxis but low or undetectable allergen-specific IgE levels must therefore be reconsidered considering their total IgE levels. Low IgE producers have been also associated with common variable immunodeficiency, lung diseases, and malignancies. A few epidemiological studies have shown a higher risk of malignancies in very low IgE producers, leading to a debated hypothesis proposing a novel, evolutionistic-relevant function for IgE antibodies for antitumor immune surveillance.

## 1. Serum IgE Levels: What Is ‘Normality’?

Immunoglobulin E (IgE) was discovered in the 1960s [1] and was soon implicated in allergies and helminthic infections [2]. Tests to measure serum IgE concentrations were developed [3,4,5] and thresholds of normality proposed [4,5,6]. However, it was soon concluded that the serum levels of total IgE in allergic and normal individuals overlap broadly [7], so that the use of this parameter in the diagnosis of allergies was discarded [8,9]. One of the targets of this review is to challenge the basis of this pessimistic conclusion, to summarize original data on serum IgE levels obtained during birth cohort studies and propose a new definition of ‘normal’ IgE levels. The clinical implications of very low total IgE levels for allergic diseases, immunodeficiencies with respiratory infections, and risk of malignancies are also discussed.

## 2. Normal Ranges of Serum IgE Levels by Exposure to Helminth Infections

The normal range of IgG serum levels is kept narrow, being limited to a three-fold factor only (from 600 to 1800 mg/dL) in humans [10]. In contrast, the average IgE levels are extremely lower and can broadly range from a few nanograms to hundreds of micrograms per milliliter of serum [11]. Moreover, opposite to the IgG serum levels, the concentration of IgE in human serum can rapidly increase even 1000-fold during helminthic infections [12] or in cystic fibrosis patients during an episode of allergic bronchopulmonary aspergillosis (ABPA) [13].

Considering that helminths are, by far, the most potent inducers of IgE [14], different ranges of normality for the total IgE are being used for helminth-free, Westernized populations (from a few to hundreds of nanograms per milliliter) [4,5] and for populations frequently exposed to helminth infections (from hundreds of nanograms per milliliter to dozens of micrograms per milliliter) [15]. Hence, the absolute levels and normality range of serum IgE is a function of exposure to helminths.

## 3. No Upper Limit and No Lower Limit in Normal Serum IgE Levels: Does It Really Mean Lack of a Normal Range?

As mentioned above, the apparent lack of constraints in the upper and lower limits of serum IgE levels greatly hampered the use of this parameter in the diagnostics of IgE-mediated allergic diseases [8,9]. On the one hand, upper limits of normal total IgE levels cannot be identified in helminth-free, Westernized populations, whose healthy individuals have IgE levels considered abnormally high but that would even be considered ‘low’ among populations living in geographic areas endemic for helminthic infections [15]. On the other hand, some healthy humans live very well with their serum IgE falling below the lowest limit of detection in IgE assays [4,16], suggesting the absence of a lower limit of normal total IgE levels [5]. The attempt to define normal ranges of total IgE levels has therefore been abandoned and the question forgotten for many decades. The ’normality’ of the serum levels of gamma-globulins, IgG1, and other immunoglobulin classes and subclasses is based on cross-sectional analyses at the population level. The above-mentioned great homogeneity of these Ig levels and their gaussian distributions [17] facilitate the identification of a ‘normal’ range. Considering that IgE levels are very heterogeneous and not normally distributed in the population, another statistical approach might be more successful in defining the normal ranges of serum tIgE levels. The following section is dedicated to observations suggesting that this is the case.

## 4. Evolution of Total IgE Levels among Westernized, Non-Allergic Children: An Analysis at Individual Level

**Figure 1:**
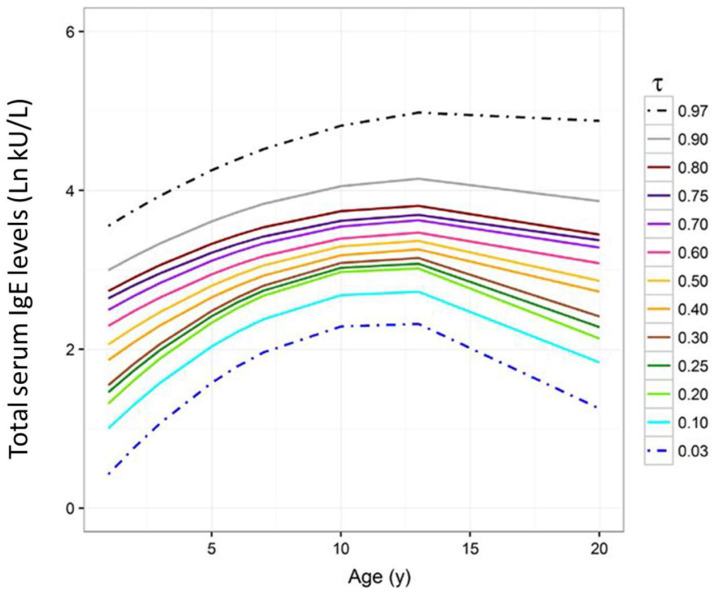
Quantile trajectories of the predicted log-transformed t-IgE levels (kU/L) by age estimated in the population of ‘never atopic’ subjects (*n* = 466) after the evaluation of the quantile regression model for longitudinal data applied considering 13 quantiles (0.03, 0.10, 0.20, 0.25, 0.30, 0.40, 0.50, 0.60, 0.70, 0.75, 0.80, 0.90, and 0.97). Quantile trajectories of the absolute t-IgE levels (log (kU/L) between two consecutive follow-ups are shown, where the values 0, 1, 2, 3, 4, 5, and 6 of the t-IgE levels in log-units (kU/L) correspond to 1.00, 2.72, 7.39, 20.09, 54.60, 148.41, and 403.43 kU/L (Adapted from [20] with permission).

The evolution of total IgE levels was examined at the individual level in the Multicenter Allergy Study (MAS), a large birth cohort born in Germany in 1990 and examined until 2020 [18]. A hallmark of this cohort study was the availability of sera from blood samples withdrawn at many time points in life, namely at 1, 2, 3, 5, 6, 7, 10, 13, and 20 years of age. In this almost unique biobank, the trajectories of the total IgE levels were first examined in children who never developed allergen-specific IgE, i.e., non-atopic or ‘normal’ children. It was confirmed that serum total IgE levels, even in non-allergic children, can differ even 100-fold [19,20] and disclosed that the trajectory of total IgE during childhood is very similar among never atopic children, independently from the absolute concentration where they are positioned [20]. A natural consequence of this novel observation was the generation of ‘growth curves’ of the total IgE levels in non-atopic children that can be represented in percentiles, similar to what is done with weight and height growth curves during childhood (Figure 1) [20]. It was therefore proposed that the normality of tIgE serum levels be defined at the individual level, according to the trajectory of the individual’s percentile on the growth chart, rather than at the population level. In other words, it was proposed to apply to the definition of the normality of tIgE levels the same criteria routinely used by pediatricians in defining normality in height and weight [20].

## 5. Defining Normality of Serum IgE Concentration at the Individual, Not at the Population, Level

By defining the normality of total serum IgE values at the individual level, the definition of ‘abnormally high’ tIgE levels would change as well. The MAS also disclosed that, at the onset of the first atopic response, a child’s trajectory deviates upwards from the ‘normal trajectory’ of the patient’s own percentile [20]. Therefore, healthy children whose trajectory of IgE levels follows the lower percentiles (e.g., <10th) may become atopic while keeping their total IgE levels within values (e.g., 80 kU/L) often considered ‘normal’ at the population level [20]. In other words, it was proposed to test the normality of the tIgE serum levels in each child with the same statistical approach routinely used by pediatricians in evaluating the ‘normality’ of a child’s weight and height on the basis of the weight and height growth charts. Unfortunately, while height and weight can be quickly measured with easy and not invasive procedures, tIgE levels can be measured only through blood drawing. Theoretically, tIgE levels should be measured in never allergic children to have a reference value to assign a percentile and evaluate whether these levels are abnormally increased when, later in life, the onset of an IgE-mediated disease is suspected [20]. Although scientifically solid enough, the feasibility of this approach is obviously limited by ethical, financial, and organizational constraints.

## 6. Total IgE Levels in Helminth-Free Populations, a Dual Genetic Control?

**Figure 2:**
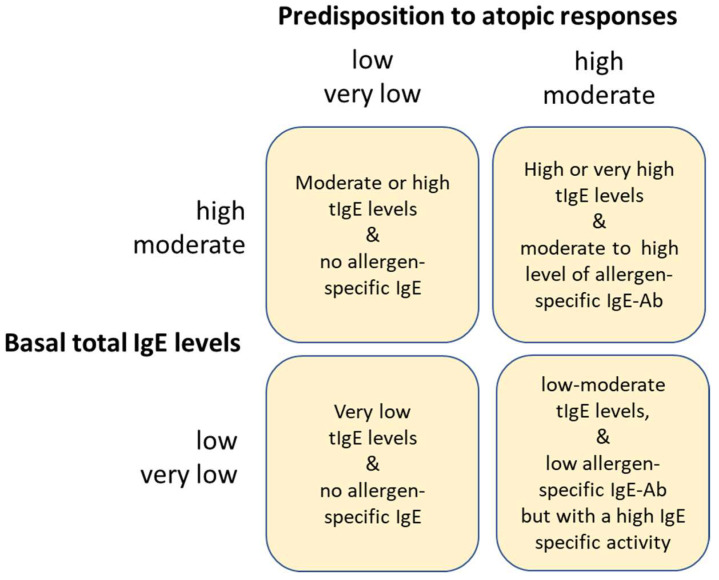
Stratification of children according to Marsh’s dual genetic model of tIgE regulation in humans. One pool of genes would control the baseline production of IgE, i.e., IgE produced in the absence of helminths and not directed against environmental allergens. A second pool of genes would be responsible for the propensity to develop IgE responses due to environmental allergens (atopy). Polymorphisms in the two pools of genes would influence the overall propensity toward ‘high’ or ‘low’ baseline IgE levels and the presence or absence of atopic IgE. The combination of these two traits would define four categories of children: low IgE producers and not-atopic, low IgE producers but atopic, high IgE producers but not-atopic, and high IgE producers and atopic.

Studies in the MAS birth cohort have also led us to speculate that, in Westernized and virtually ‘helminth-free’ children, tIgE levels derive largely from two distinct pools: IgE spontaneously produced (‘normal’, baseline IgE) and IgE produced during atopic responses to environmental allergens (‘abnormal’ or atopic IgE) [19,20]. The biological plausibility in this hypothesis comes from genetic studies that distinguished between genes contributing to ‘basal’ IgE levels [21] and genes contributing to atopic IgE [22]. By oversimplification, we may therefore categorize children into four subgroups, according to their genetic predisposition toward a low or high production of ‘normal’ IgE and the independent genetic predisposition toward abnormal or atopic IgE responses (Figure 2). A dual genetic control of IgE production would also contribute to explaining our difficulties in defining the ‘normal’ ranges of total serum IgE with a traditional approach based on a statistical evaluation of cross-sectional data at the population level.

## 7. The Very Low IgE Producer but Atopic Patient: An Intriguing and Difficult Clinical Phenotype

The concepts exposed so far may be of help in understanding those infrequent, but not rare, clinical cases of patients with IgE-mediated allergic manifestations but very low allergen-specific IgE levels. This category includes the patients with no genetic predisposition toward producing high levels of IgE but with a genetic predisposition toward producing allergen-specific IgE responses (Figure 2). According to the model presented here, the total IgE levels of these patients, before developing their allergic disease, belong to the lowest percentiles of the growth chart. When they start producing allergen-specific IgE, their total IgE levels remain within the population normal range and their specific IgE levels stay very low, even below 1 kU/L. Case reports have described children undergoing anaphylactic shock after milk [23] ingestion notwithstanding very low allergen-specific IgE-Ab levels but combined with low total IgE levels or patients with a peanut allergy with a positive basophil activation test but very low peanut-specific IgE antibodies [24]. The relevance of a high specific activity in predicting an allergy to peanuts, together with other parameters such as the sIgE antibody levels, has been recently confirmed [25]. Other studies examined young adults with serious allergies to cats and vanishingly low IgE antibody titers [26] (Table 1).

IgE-specific activity is one of the four crucial parameters for the effective degranulation of mast cells and basophils [27]. It is speculated that IgE molecules not recognizing the culprit allergen reduce the probability that two allergen-specific molecules are bridged by the allergen itself [27]. Hence, the total IgE levels and the specific activity of IgE antibodies toward the suspected allergen should always be measured before excluding an allergy based on ‘low’ levels of specific IgE antibodies [20]. In other words, when we observe a discrepancy between the low levels of allergen-specific IgE and clear-cut allergic reactions to that allergen, it is essential to exclude that the patient is a ‘very low IgE producer’ and therefore a candidate for IgE responses with a high specific activity. Accordingly, predictive models for anaphylaxis to peanuts suggest incorporating tIgE levels as essential parameters for a correct diagnosis [25,27,28]. However, not only IgE but also IgG antibodies may play a relevant role in regulating the pathogenetic impact of IgE antibodies, as previously discussed [25].

## 8. Patients with ‘Local Allergic Rhinitis’: Are They ‘Low Total IgE Producers’?

‘Local Allergic Rhinitis’ (LAR) has been defined as a form of allergic rhinitis, confirmed by Nasal Allergen Provocation Tests (NAPT) in patients with no positive skin prick test (SPT) or IgE test against the offending allergen [29,30]. The real prevalence of LAR among patients with chronic, non-infectious rhinitis has been hotly debated, ranging from over 25% in some studies [30] to less than 1% in others [31]. In the initial stages of allergic rhinitis, IgE are produced only at the mucosal level, and a small portion spills over into the circulation [32]. Under these circumstances, a negative outcome of allergen-specific SPT and IgE assay may be associated with a positive NAPT and symptoms of allergic rhinitis upon allergen exposure [32]. Moreover, recent studies in patients with LAR have found allergen-specific IgE antibodies absorbed on the surface of circulating basophils in the absence of allergen-specific IgE antibodies in the patient’s serum [33]. It is proposed that, at first, basophils, through their Fc-epsilon receptors, can capture all the very few IgE antibodies that spilled over from the nasal mucosa into the circulating blood and that, at a later stage, IgE tests can detect free allergen-specific IgE antibodies in the serum, i.e., only when their mucosal production and spillover process is advanced enough and the IgE-binding capacity of circulating basophils Fc-epsilon receptors is saturated [33]. In the first stage of allergic rhinitis, the concentration of free IgE-Ab in the serum would be too low to be detected with the currently available commercial IgE assays [33]. In keeping with the model presented here, it may be hypothesized that this initial negative SPT and IgE test stage of allergic rhinitis may last longer and even be permanent in very low IgE producers [31]. Interestingly, when these patients are treated with monoclonal anti-IgE antibodies, allergen-specific IgE antibodies become detectable in the serum [33], which is consistent with the hypothesis that Omalizumab binds IgE antibodies as soon as they penetrate the circulation and prevents their binding to the Fc-epsilon receptors of the circulating cells [33]. Increased IgE antibody levels following Omalizumab is a more general phenomenon also observed in patients with high specific IgE antibody levels [34]. The hypothesis that patients with ‘true’ LAR are also low or very low total IgE producers deserves to be explored.

## 9. IgE Immunodeficiency: Does It Exist?

The existence of a deficiency in immunoglobulin E production has been hotly debated since the discovery of this Ig class. Given their very low levels, very close to the lower limit of detection of most immunoassays, it has been difficult to find an operative definition for such an immunodeficiency [34]. Levels below 2 kU/L or 2.5 kU/L and other cut-off points have been proposed as the definition of IgE deficiency [34]. Very low IgE values are not considered by most doctors as bad news, also reflecting the common opinion that IgE, although contributing to fighting off many parasite infestations, are not essential in our quite redundant immune system [35] and that people with undetectable IgE are not allergic [4].

A possible link between low or undetectable IgE levels and primary immunodeficiencies, with a particular focus on antibody deficiencies, was soon suspected after the discovery of IgE themselves [36]. IgE levels varied among patients with selective IgA deficiency, and a combined deficiency did not necessarily predispose them to recurrent infections [36]. Nevertheless, very low IgE levels are also frequent in subjects with common variable immunodeficiency (CVID) [37]. Testing for the total IgE may even be a good strategy in suspecting or disclosing otherwise unrecognized immune defects [38], such as CVID [16]. In an immunodeficiency and allergy clinic, low IgE levels were associated with low IgG3 and IgG4 in allergy referrals and with lower IgG1, IgG2, and IgG4 levels in immunodeficiency referrals [39]. Undetectable serum IgE levels (<2 kU/L) but not very high IgE levels were very frequent (75.6%) in a cohort of 354 patients with CVID [40]. 

There is still no report of genetic defects leading to isolated IgE deficiency (i.e., associated with normal levels of other Ig classes and subclasses). However, a family with a cluster of cases of IgE deficiency, associated with chronic bronchitis and fibrotic lung disease, has been reported [41]. Beyond the above-reported discussion on terminologies and disease definitions, undetectable or very low serum total IgE levels expose patients to diseases or symptoms including recurrent respiratory infections, autoimmune diseases, and airway diseases [35] (Table 1).

Whether these associations are genuine or spurious, i.e., due to parallel defects in other immunoglobulin classes, remains a matter of debate [35]. In a series of 44 patients with IgE levels lower than 2.5 kU/L, of which 57% had depressed serum levels of other immunoglobulins, arthralgias, chronic fatigue, and symptoms suggestive of airway infection, autoimmune diseases were more frequent than among patients with IgE levels higher than 2.5 kU/L [42]. Interestingly, these differences were observed among both patients with selective IgE deficiency and patients with IgE deficiency complicated by deficits in other immunoglobulin classes [42]. The frequency of chronic sinopulmonary inflammatory diseases, chronic rhinosinusitis, asthma, and COPD was not different among patients with respiratory diseases and very low or very high serum tIgE levels [43]. Among children and adults with tIgE levels below 2 kU/L, a higher prevalence of asthma and hyperreactive airways disease was diagnosed in children, while both children and adults were more frequently affected by chronic sinusitis, otitis media, autoimmune, and oncological diseases than their respective controls [44]. A systematic investigation on the biological basis, pathogenetic mechanisms, and epidemiological and clinical aspects of humans with no or very low levels of IgE has never been done. Until then, the debated issue of whether an IgE immune deficiency exists will not be solved.

## 10. Allergo-Oncology: Are Allergic Patients at Lower Risk of Malignancies?

The evolutionistic origin of IgE is extremely old, dating back several hundred million years [45], suggesting that IgE-mediated anaphylactic mechanisms are fundamental. The anaphylactic function of IgE is dependent on Fc-epsilon receptor I molecules (CD23) on the surfaces of the degranulating cells and the IgE master with Fc-epsilon receptor I as a complex biological system that has also been called the ‘IgE-network’ [46]. This system provides a defense mechanism against infections and toxins [47], but a consensus on whether this is the most relevant function of this Ig isotype has never been reached.

Multiple epidemiological studies have described inverse associations between allergic diseases and malignancies [48,49] and led to the hypothesis—supported by experimental studies on animal models [50]—that IgE may be involved in antitumor surveillance, an area of investigation purposedly denominated ‘allergo-oncology’ [51]. In the largest of these epidemiological studies, among 1,102,247 US men and women who were cancer-free at baseline, with 18 years of follow-up from 1982 to 2000, a total of 81,114 cancer deaths were registered. A very weak but significant inverse association between a history of both asthma and hay fever and overall cancer mortality and colorectal cancer mortality in comparison with persons with neither asthma nor hay fever was observed [52]. In the same study, hay fever and asthma were associated with a significantly lowered risk of pancreatic cancer mortality and leukemia mortality, respectively [52]. Similarly, a lower risk of developing cancer was found among hospitalized patients discharged with a diagnosis of asthma [53]. By contrast, a 10-year follow-up analysis on 93% of the 6913 adults participating in the First National Health and Nutrition Examination Survey (NHANES-I) showed that an allergic history weakly but significantly increased the risk of subsequent malignancy, with hives and lymphatic–hematopoietic being the most frequently involved allergic and malignant phenotypes [54]. Many other cross-sectional, longitudinal, or case–control epidemiological studies investigated the association of allergies and cancer, but no definitive conclusion on an inverse epidemiological association has been reached.

Nevertheless, it has been proposed that FcεRI, whose expression is upregulated by IgE, mediate through IgE themselves antibody-dependent cellular phagocytosis (ADCP) and antibody-dependent cellular cytotoxicity (ADCC) against tumor cells which membrane antigens are recognized by IgE antibodies [55]. According to allergo-oncologists, these IgE antibodies recognizing tumor-associated antigens might activate different categories of antitumor effector cells, including macrophages, mast cells, and eosinophils [51]. Tumor-specific IgE have been induced in BALB/c mice with a mimotope vaccine orally administered under anti-acidic conditions, and these antibodies proved to be functional against breast cancer cells [51]. These outcomes led to proposing protocols to transform tumor-associated antigens in allergens, orally induce IgE antibodies, and activate strong antitumor ADCP and ADCC responses [51].

## 11. Low IgE Producers: Are They at Risk of Malignancies?

If IgE antibodies really do participate in immune surveillance mechanisms against tumor cells, then low IgE producers should be at risk of developing malignancies. Indeed, a few reports in the last decade have associated IgE deficiency with malignancies. Among 63 patients with IgE deficiencies, a significantly high rate of prior malignancy was found (21 out of 63, 33%) compared to those without IgE deficiencies, independently from the presence of CVID [56]. In a larger epidemiological study on the 2005–2006 National Health and Nutrition Examination Survey, the rate of prior malignancy, including breast, skin, uterine, cervical, lung, prostate, and hematologic cancer, was significantly higher in the IgE-deficient group (here defined as IgE levels below 2.5 kU/L) compared with individuals with high and very high IgE levels [57]. Causality in the relationship of low or deficient IgE levels with malignancies was not proven in these retrospective studies. However, 34 IgE-deficient patients with no malignancy diagnosis, identified in 2014–2015 and monitored for an average period of 43.5 months, showed incidences of malignancy significantly higher than non-IgE-deficient controls recruited during the same period/from the same hospital, and this outcome was independent from a diagnosis of CVID [58]. In line with these observations, it has also been speculated that a very low IgE level may be a novel early biomarker predicting cancer development, and it has been suggested that IgE-deficient patients [51] should be regularly monitored for malignancy [51,58] Any conclusion on this intriguing hypothesis is premature, and many other longitudinal studies will have to ascertain a causative link between very low or undetectable IgE levels and malignancies.

## 12. Perspectives

Five decades of investigation on IgE and allergic diseases have been mostly dedicated to investigating the pathologic effect of having too many of these immunoglobulins. For a few years now, scientists and clinicians have also been studying the other side of the coin. These investigations are opening new perspectives not only on the understanding of ‘low-IgE-producers’ and allergic patients but also on the search for the still-elusive function and evolutionistic meaning of this intriguing antibody.

## Figures and Tables

**Table 1:** Diseases reported to be more frequent among humans with very low total IgE serum levels (<2.5 KU/L) *.

1. Low serum level of other Ig isotypes, CVID
2. Recurrent, chronic airways infections
3. Autoimmune diseases
4. Malignancies
5. Local Allergic Rhinitis

* *Insufficient evidence to reach consensus*.

## Data Availability

Not applicable.
